# Prevalence and characteristics of flavoured tobacco use among students in grades 10 through 12: a national cross-sectional study in Canada, 2012–2013

**DOI:** 10.1186/s12971-017-0124-0

**Published:** 2017-03-24

**Authors:** Yelena Bird, Jennifer May, Chijioke Nwankwo, Razi Mahmood, John Moraros

**Affiliations:** 1 School of Public Health, University of Saskatchewan, 104 Clinic Place, E-Wing Health Sciences, Room 3322, Saskatoon, SK S7N 2Z4 Canada; 2 Health Promotion, Lung Association of Saskatchewan, Saskatoon, Canada

**Keywords:** Flavoured tobacco, Adolescents, Canada

## Abstract

**Background:**

Tobacco use is the leading cause of preventable death in Canada and the world. Despite documented decreases in the prevalence of smoking in Canada, increases in flavoured tobacco use by its youth poses a serious public health concern. This study examined the prevalence and characteristics of flavoured tobacco use among a national sample of Canadian students in grades 10 through 12.

**Methods:**

This study used a cross-sectional design on a nationally generalizable, school-based, Youth Smoking Survey (YSS), 2012–2013. It incorporated data from a representative sample of 19,979 students in grades 10–12 from across Canada. Univariate and multivariate logistic regression models were used to examine differences in flavoured tobacco use (menthol cigarettes, flavoured little cigar or cigarillo, flavoured cigar, flavoured tobacco in water pipe [hookah]) by demographic (sex, grade and ethnicity) and social characteristics (friends, siblings, parents/guardians who are smokers and weekly personal spending money).

**Results:**

This study found that 14.8% of the participating students used flavoured tobacco in the past 30-days. Results of the logistic regression analysis show that flavoured tobacco use was significantly higher among male students [(OR = 1.63; 95% CI = 1.36–1.95)], who had at least one friend or sibling who smoke [(OR = 2.20; CI = 1.62 to 2.99) and (OR = 1.51; CI = 1.22 to 1.88), respectively] and who received greater than $20/week in personal spending money [(OR = 1.76; CI = 1.26 to 2.45)].

**Conclusions:**

The results of our study indicate that flavoured tobacco use is a growing public health concern and has a strong appeal among youth in Canada. This is a particularly troubling finding, especially in light of the fact that there is a national ban on certain flavoured tobacco products. To be effective, strategies specifically tailored for youth using flavoured tobacco would require appropriate educational/prevention initiatives, more comprehensive legislation and better regulatory mechanisms.

## Background

Tobacco use is the leading cause of preventable death in Canada and the world [1]. It has been associated with a range of medical conditions known to cause significant morbidity and mortality [1]. Current global estimates of the mortality from tobacco stand at 6 million deaths annually [2]. Since the 1990’s, intense public health efforts have concentrated in reducing the burden of disease resulting from tobacco. These actions have effectively contributed to a steady decline of smoking rates over time [3, 4]. In Canada, it is estimated that the prevalence of smoking is currently at its lowest point in nearly two decades [4] and the average number of cigarettes smoked daily has decreased by more than 2 cigarettes since 1999 [3].

These encouraging trends are threatened by the documented growing appeal of flavoured tobacco (menthol cigarettes, flavoured little cigar or cigarillo, flavoured cigar, flavoured tobacco in water pipe [hookah]) among youth [5, 6]. It has been reported that approximately 2 of every 5 current youth smokers (approximately 126,000 young Canadians), use flavoured tobacco [7]. These tobacco products come in flavours that appeal to youth including vanilla, chocolate, bubblegum, watermelon, cherry, and strawberry. They are packaged in enticing colours aimed at youth and carry no health warnings. They are associated with less throat and upper respiratory tract irritation, which makes it easier to start and continue to smoke them and exposes youth to the long term effects of nicotine [8]. This puts youth at risk to become addicted and establish use patterns that persist into adulthood, making them life-long tobacco consumers [9].

Flavoured tobacco products have been falsely advertised as possible aids for smoking cessation and harm reduction by the tobacco industry [10]. Thus, it is not surprising to find that most young flavoured tobacco users believe them to be less harmful than regular cigarettes [11–13]. Similarly, one third of adults sampled in a US study believed the harmful effects of flavoured tobacco to be minimal [14]. These erroneous beliefs are fundamentally premised on an effective but misleading marketing campaign by the tobacco industry [15] and stand in stark contrast to the mounting evidence in the literature that details a number of deleterious effects attributed to the use of flavoured tobacco [16–18].

In Canada, concerns over the health effects of flavoured tobacco products [5, 19] along with their rise in popularity among youth [20] led the Canadian government to implement Bill C-32 in July 2010 [21]. Bill C-32 prohibits the sale of cigarettes, little cigars and cigarillos, and blunt wraps that weigh less than 1.4 g and contain flavouring agents (excluding menthol). However, Bill C-32 does not cover all tobacco products and many manufacturers simply increased the weight of their products to more than 1.4 g to circumvent the law. Thus despite the national ban, it has been reported that more than half of high school students in Canada who smoke, use flavoured tobacco [6].

Flavoured tobacco use among Canadian youth is the result of a set of complex and dynamic interactions between youth and their social environment. However, the interactions between their demographic (sex, grade and ethnicity) and social characteristics (friends, siblings, parents/guardian who are smokers and weekly personal spending money) have not been sufficiently studied. The present study aims to use a large, nationally representative sample in order to identify the prevalence and characteristics associated with the use of flavoured tobacco among Canadian students in grades 10 through 12.

## Methods

### Study design

This study used a cross-sectional, stratified design on a nationally generalizable, school-based Youth Smoking Survey (YSS) in 2012–2013. Stratification was based on the health region smoking rates (as determined by the Canadian Community Health Survey data), the participating school’s postal code and the population size of major metropolitan areas [22]. The survey design and sample weights allow for population-based estimates and have been explained in detail elsewhere [23].

### Participants

The 2012–2013 YSS involved a total of 38,667 student participants from grades 7–12 across 450 schools in Canada. Schools from the province of Manitoba and the Northwest, Nunavut and Yukon territories as well as students living in institutions or on First Nations reserves did not participate in the survey. Research has shown that students in lower grades (7–9) differ significantly from students in higher grades (10–12) with regard to their behaviours and a variety of measures relating to tobacco use [24]. Our study focused on students in grades 10–12 (*n* = 19,979).

### Variables explored

#### Outcome variable

The outcome variable was current flavoured tobacco use (past 30-day use). The outcome variable was derived from a question in the YSS asking: “In the last 30-days, did you use any of the following flavoured tobacco products?” Responses included: “menthol cigarettes (Yes/No), flavoured little cigar or cigarillo (Yes/No), flavoured cigar (Yes/No), flavoured tobacco in water pipe (hookah) (Yes/No).” Students who answered yes to any of these four questions were classified as being current flavoured tobacco users.

#### Independent variables

The independent variables of interest included the respondent’s demographic (sex = male/female; ethnicity = White, Black, Asian, Aboriginal, Hispanic; grade = 10–12) and social characteristics (how many of the respondent’s friends, siblings and parents/guardians are smokers = 0, 1, 2, 3 or more; weekly personal spending money = $0 to > $100).

### Data analysis

The survey was weighted by the data provider in order to adjust for differential response rates across groups and to allow for generalization of the findings to the Canadian student population [22]. Our study used descriptive analysis to summarize the basic features of the data, looking at frequencies and distributions of the outcome and independent variables of interest. Univariate analysis was conducted to determine the crude association between each of the independent variables (sex, grade, ethnicity, number of friends, siblings, and parents/guardians who smoke and weekly personal spending money) and the outcome variable of interest (flavoured tobacco use). Logistic regression modelling was carried out using the PROC SURVEYLOGISTIC command to examine the relationship between flavoured tobacco use and the independent variables of interest.

Assumptions of logistic regression were checked, and backwards elimination strategy was used, when developing the models. When variables were removed, confounding was assessed at each stage. If there was a change in the effect estimate of the primary predictor by 20% or more with the inclusion of a variable, the variable would be retained in the model as a confounder. All possible two-way interactions involving the predictor variable and the variables of interest were assessed using a *p*-value of 0.05 for statistical significance. All analysis was carried out using SAS version 9.3.

## Results

In 2012–2013, 14.8% of the participating students from grades 10–12, self-identified as having used flavoured tobacco over the past 30-days in Canada. Past 30-day use of flavoured tobacco as a percentage was highest among males (18.8%), who attended 12^th^ grade (17.4%), and who self-reported being Aboriginal (23.9%), Hispanic (21.3%) and Black (19.0%). Also, youth who used flavoured tobacco products in the past 30-days, reported having one or more friends who smoked (68.3%), one or more siblings who smoked (91.2%), and one or more parents/guardians who smoked (63.9%). In terms of their weekly personal spending, 23.3% reported having greater than $100 per week to spend on themselves, while 8.7% reported having no personal weekly monies (Table 1).

**Table 1:** Descriptive characteristics of the study population among 10–12 grade school students in Canada, 2012–2013

Variables		Flavoured Tobacco Use (Past 30 days)	No Flavoured Tobacco Use (Past 30 days)	Total (N)
Outcome Variable				
Flavoured Tobacco Use (Past 30 days)		14.77%	85.23%	19979
Independent Variables				
Sex (*n* = 19979)	Male	18.8%	81.2%	9808
	Female	10.9%	89.1%	10171
Grade	Tenth	12.7%	87.3%	7544
	Eleventh	15.0%	85.0%	6982
	Twelfth	17.4%	82.6%	5453
Ethnicity (*n* = 18818)	White	14.5%	85.5%	14389
	Black	19.0%	81.0%	814
	Asian	7.5%	92.5%	1942
	Aboriginal	23.9%	76.1%	1270
	Hispanic	21.3%	78.7%	403
Friend Smokers (*n* = 18048)	None	4.6%	95.5%	9861
	One	12.1%	87.9%	2278
	Two	19.7%	80.3%	1571
	Three or more	36.5%	63.5%	4338
Sibling Smokers (*n* = 18141)	None	11.0%	89.0%	14149
	One	23.2%	76.8%	2763
	Two	25.9%	74.1%	718
	Three or more	42.1%	57.9%	511
Parent/Guardian Smokers (*n* = 18921)	None	10.4%	89.6%	10935
	One	16.5%	83.5%	4284
	Two	20.1%	79.9%	2107
	Three or more	27.3%	72.7%	1595
Weekly Personal Spending Money (*n* = 16546)	$0	8.7%	91.3%	2783
	$1–$5	12.5%	87.5%	869
	$6–$10	10.3%	89.7%	1199
	$11–$20	12.6%	87.4%	2594
	$21–$40	17.1%	82.9%	2639
	$40–$100	16.7%	83.3%	2733
	>$100	23.3%	76.7%	3729

Univariate analysis suggests that sex (*p* < 0.0001), grade (*p* < 0.0003), ethnicity (*p* < 0.0001), friends who smoke (*p* < 0.0001), siblings who smoke (*p* < 0.0001), parents/guardians who smoke (*p* < 0.0001) and weekly personal spending money (*p* < 0.0001) were significantly associated with flavoured tobacco use among students in grades 10–12. Odds ratios are shown in relation to a reference category for each variable (Table 2).

**Table 2:** Univariate analysis of flavoured tobacco use among 10–12 grade school students in Canada, 2012–2013

Variables		Odds Ratio (95% CI: Lower to Upper)	*p*-value (α = 0.05)
Sex (Ref = Female)	Males	1.73 (1.48 to 2.02)	<0.0001
Grade (Ref = Grade 10)	Grade 11	1.20 (1.00 to 1.44)	0.0003
	Grade 12	1.53 (1.24 to 1.87)	
Ethnicity (Ref = White)	Black	0.83 (0.58 to 1.19)	< 0.0001
	Asian	0.53 (0.39 to 0.71)	
	Aboriginal	2.17 (1.74 to 2.70)	
	Hispanic	1.49 (1.03 to 2.17)	
Friend Smokers (Ref = None)	1	2.82 (2.05 to 3.88)	< 0.0001
	2	5.28 (3.82 to 7.30)	
	3 or more	10.47 (8.56 to 12.81)	
Sibling Smokers (Ref = None)	1	2.82 (2.29 to 3.48)	< 0.0001
	2	3.43 (2.59 to 4.54)	
	3 or more	7.07 (5.01 to 9.97)	
Parent/Guardian Smokers (Ref = None)	1	1.76 (1.44 to 2.15)	< 0.0001
	2	2.25 (1.82 to 2.79)	
	3 or more	3.63 (2.80 to 4.69)	
Weekly Personal Spending Money (Ref = Zero)	$1–$5	1.05 (0.72 to 1.53)	< 0.0001
	$6–$10	1.16 (0.81 to 1.68)	
	$11–$20	1.73 (1.28 to 2.34)	
	$21–$40	2.33 (1.75 to 3.09)	
	$41–$100	2.23 (1.65 to 3.00)	
	> $100	3.76 (2.93 to 4.81)	

Flavoured Tobacco Use = 1 if: Menthol cigarette or Flavoured little cigar or cigarillo or Flavoured cigar or Flavoured tobacco in a water-pipe (hookah) was used in the past 30 days

Flavoured Tobacco Use = 0 if: I did not use any of these things (above) in the past 30 days

Multivariate logistic regression analysis examined the odds of being a flavoured tobacco user. The results found that male students (OR = 1.63, 95% CI: 1.36 to 1.95), who had at least one friend (OR = 2.20, 95% CI: 1.62 to 2.99) or sibling (OR = 1.51, CI: 1.22 to 1.88) who smoke and who received greater than $20/week in personal spending money (OR = 1.76; CI = 1.26 to 2.45) were significantly associated with using flavoured tobacco when compared to students who did not (Table 3).

**Table 3:** Factors associated with flavoured tobacco use by logistic regression analyses among 10–12 grade school students in Canada, 2012–2013

Variables		Odds Ratio (95% CI: Lower to Upper)	*p*-value (α = 0.05)
Sex (Ref = Female)	Males	1.63 (1.36 to 1.95)	< 0.0001
Ethnicity (Ref = White)	Black	0.94 (0.60 to 1.45)	0.0470
	Asian	0.65 (0.46 to 0.92)	
	Aboriginal	1.19 (0.88 to 1.60)	
	Hispanic	1.32 (0.84 to 2.08)	
Friend Smokers (Ref = None)	1	2.20 (1.62 to 2.99)	< 0.0001
	2	3.80 (2.71 to 5.31)	
	3 or more	7.08 (5.66 to 8.86)	
Sibling Smokers (Ref = None)	1	1.51 (1.22 to 1.88)	< 0.0001
	2	1.55 (1.09 to 2.20)	
	3 or more	3.40 (2.33 to 4.96)	
Weekly Personal Spending Money (Ref = Zero)	$1–$5	1.05 (0.68 to 1.62)	< 0.0001
	$6–$10	1.12 (0.71 to 1.76)	
	$11–$20	1.23 (0.87 to 1.76)	
	$21–$40	1.76 (1.26 to 2.45)	
	$41–$100	1.62 (1.17 to 2.26)	
	> $100	2.46 (1.82 to 3.33)	

Flavoured Tobacco Use = 1 if: Menthol cigarette or Flavoured little cigar or cigarillo or Flavoured cigar or Flavoured tobacco in a water-pipe (hookah) was used over the past 30 days

Flavoured Tobacco Use = 0 if: I did not use any of these things (above) in the last 30 days

The variables of “friends who smoke” showed a graded association of a student using flavoured tobacco as the number of friends who smoke increased from one (OR = 2.20, 95% CI: 1.62 to 2.99) to two friends (OR = 3.80, 95% CI: 2.71 to 5.31) and three or more friends who smoke (OR = 7.08, 95% CI: 5.66 to 8.86), when compared to students who reported having no friends who smoke. A similar pattern was seen with siblings who smoke, as individuals who had one sibling (OR = 1.51, 95% CI: 1.22 to 1.88), two siblings (1.55, 95% CI: 1.09 to 2.20) and three or more siblings who smoke (OR = 3.40, 95% CI: 2.33 to 4.96) demonstrated a graded association with flavoured tobacco use, when compared to students with no siblings who smoke (see Table 3). There were no statistically significant associations with parent/guardian smokers or by high school grade (grade 10, 11, 12) and a student using flavoured tobacco.

## Discussion

This study sought to determine the prevalence and characteristics associated with the use of flavoured tobacco among a national sample of Canadian students in grades 10 through 12. It provides insight and adds knowledge to our understanding of flavoured tobacco use among youth in Canada along two discrete but interrelated perspectives (demographic and social).

From a demographic perspective, our study found that 14.8% of the students reported using flavoured tobacco. This is similar to the prevalence reported among high school students in the US [25] and previous studies in Canada [6, 26]. Flavoured tobacco is becoming increasingly popular among students as evidenced by the fact that one out of every three Canadian youth has tried it [7] and sales have increased by eightfold in six years [21]. This may be a reflection of the widespread belief by youth that flavoured tobacco is a better alternative to regular tobacco, less harmful to their health and more fun to use [15, 27]. Our findings also demonstrate a progressive rise in flavoured tobacco use with increasing grades 10–12 among Canadian students (16–18 years old). This is a period of identity development, increased curiosity and risk taking behaviour [28]. The tobacco industry, exploits the inherent vulnerability of this transitional period by focusing their trendy marketing and advertising campaigns on themes that resonate with youth such as independence, sophistication, fun and rebellion [9].

In our study, male students were significantly more likely to use flavoured tobacco compared to females. This was consistent with evidence reported elsewhere in the literature [11–13]. It is possible that male students have higher risk taking proclivities/behaviours along with easier access and higher affordability levels to flavoured tobacco when compared to females. Students who self-identified as Aboriginal or Hispanic had increased odds of using flavoured tobacco when compared to White students but these differences were not statistically significant. Our results are corroborated by several Canadian studies that show an increased burden of smoking among Aboriginal youth [29, 30]. However, there is not much in the literature detailing the smoking situation of Hispanic youth in Canada. This is an area that requires further study as evidence from Mexico [31, 32] and the US [9, 11] shows that Hispanic youth are at an increased risk for smoking behaviours, including the use of flavoured tobacco [33].

From a social perspective, we found a significant association between the number of siblings or friends who smoke and the likelihood of a student using flavoured tobacco after adjusting for all other covariates. Interestingly, our study found peers to have a more pronounced influence on students’ use of flavoured tobacco when compared to their siblings and even their parents/guardians. There are multiple mechanisms that may help explain this finding. It may be the result of the increased amount of time youth spend with their peers [34] and the opportunities for social learning [35], ease of access to flavoured tobacco [35–37] and direct pressures to identify, bond and gain their respect and acceptance at this critical time of their development [38].

Finally, we found that the more money a student had available to spend in a week, the higher the likelihood of them using flavoured tobacco. Several studies have associated youth’s access to spending money with their risk of smoking [6, 39, 40]. A study in Ontario, Canada, found that even small differences in weekly spending allowance had an incremental and significant impact on the smoking status among youth. It reported that students who had less than $10 per week were significantly less likely to be smokers, those with $20 per week were significantly more likely to be experimental smokers, and students with more than $30 per week were significantly more likely to be current smokers [40].

### Strengths and limitations

Flavoured tobacco use as defined for the purposes of this study represented use in the past 30-days. This time limitation may not allow us to adequately determine the volume or frequency of use. Secondly, there were no objective measures of flavoured tobacco use since all the measures in the study were self-reports. Third, the YSS is a school-based survey and therefore, youth who do not attend school were excluded from our sample. Lastly, the study used of a cross-sectional design and therefore, it is unable to draw causal inferences between the variables studied and flavoured tobacco use. Despite the aforementioned limitations, this study also has a number of significant strengths. It used a national sample that was large in size and representative in scope and specifically looked to identify the prevalence and demographic and social characteristics in the use of flavoured tobacco among Canadian students in grades 10–12.

## Conclusions

The results of our study show that flavoured tobacco use is a growing public health concern and has a strong appeal among youth in Canada. This is a particularly troubling finding, especially in light of the fact that there is a national ban on certain flavoured tobacco products. To be effective, strategies specifically tailored for youth using flavoured tobacco would require appropriate educational/prevention initiatives, more comprehensive legislation and better regulatory mechanisms. These interventions are urgently needed in order to prevent erosion and safeguard the significant gains made in our efforts to reduce smoking rates in Canada.

## Abbreviations

US: United States
YSS: Youth smoking survey
